# The effects of extracellular vesicles derived from Krüppel-Like Factor 2 overexpressing endothelial cells on the regulation of cardiac inflammation in the dilated cardiomyopathy

**DOI:** 10.1186/s12951-022-01284-1

**Published:** 2022-02-09

**Authors:** Wenfeng Zhang, Ziwei Chen, Shuaihua Qiao, Siyuan Chen, Hongyan Zheng, Xuan Wei, Qiaoling Li, Biao Xu, Wei Huang

**Affiliations:** 1 Department of Cardiology, Nanjing Drum Tower Hospital Clinical College of Nanjing Medical University, Nanjing, 210008 China; 2 Department of Cardiology, Affiliated Drum Tower Hospital, Medical School of Nanjing University, Nanjing, 210008 China

## Abstract

**Background:**

Dilated cardiomyopathy (DCM) is one of the common causes of heart failure. Myocardial injury triggers an inflammatory response and recruits immune cells into the heart. High expression of Krüppel-like factor 2 (KLF2) in endothelial cells (ECs) potentially exerts an anti-inflammatory effect. However, the role of extracellular vesicles (EVs) from KLF2-overexpressing ECs (KLF2-EVs) in DCM remains unclear.

**Methods and results:**

EVs were separated from the supernatant of KLF2-overexpressing ECs by gradient centrifugation. Mice were repeatedly administered low-dose doxorubicin (DOX) and then received KLF2-EVs through an intravenous injection. Treatment with KLF2-EVs prevented doxorubicin-induced left ventricular dysfunction and reduced the recruitment of Ly6^high^ Mo/Mø in the myocardium. We used flow cytometry to detect Ly6^high^ monocytes in bone marrow and spleen tissues and to elucidate the mechanisms underlying this beneficial effect. KLF2-EVs increased the retention of Ly6C^high^ monocytes in the bone marrow but not in the spleen tissue. KLF2-EVs also significantly downregulated C–C chemokine receptor 2 (CCR2) protein expression in cells from the bone marrow.

**Conclusions:**

EVs derived from KLF2-overexpressing ECs reduced cardiac inflammation and ameliorated left ventricular dysfunction in DCM mice by targeting the CCR2 protein to inhibit Ly6C^high^ monocyte mobilization from the bone marrow.

**Graphical Abstract:**

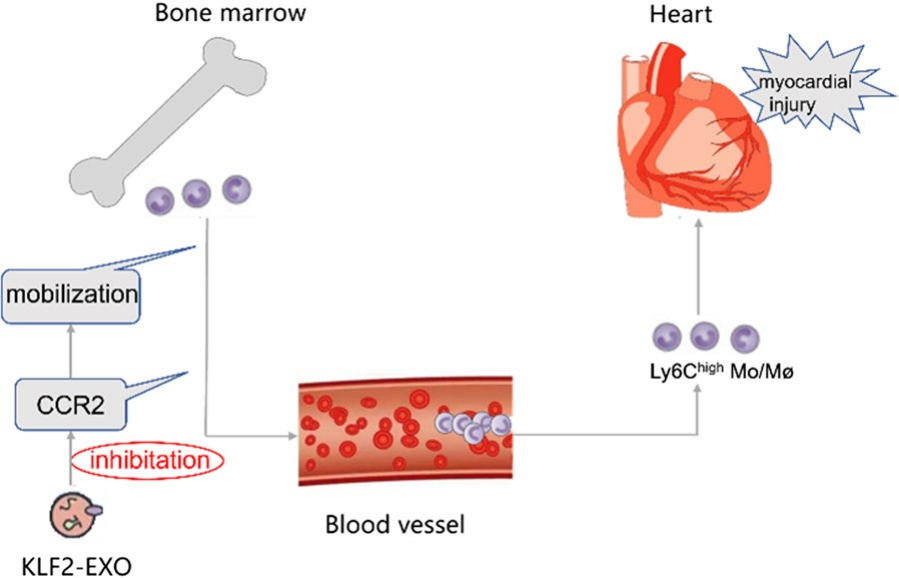

**Supplementary Information:**

The online version contains supplementary material available at 10.1186/s12951-022-01284-1.

## Background

Dilated cardiomyopathy (DCM) is a disease characterized by progressive worsening of contractile dysfunction and ventricular dilation [1–4]. To date, the 5-year survival rate of patients with DCM is less than 50% without specific treatment [5]. Therefore, studies aiming to explore therapeutic strategies for DCM are crucial. Recently, several studies have indicated that the inflammatory response and immune cells play important roles in the process of DCM [6–8]. However, the exact pathogenesis of DCM remains unclear.

The endothelial cells (ECs) lining all blood vessels are involved in many physiological processes, such as homing immune cells to specific sites in the body, and function as critical regulators of tissue homeostasis [9–11]. In previous studies, KLF2 was expressed at high levels in ECs under laminar flow conditions, which showed an anti-inflammatory phenotype [11, 12]. Under turbulent flow, KLF2 was expressed at low levels in ECs, which showed a proinflammatory phenotype. Previous studies from our group revealed that KLF2-overexpressing ECs effectively preserve the anti-inflammatory phenotype and contribute to regulating immunity by secreting extracellular vesicles (EVs) [13].

EVs, small vesicles with a diameter of approximately 40–150 nm, are secreted by different living cells, such as ECs, macrophages, mesenchymal stem cells, lymphocytes, and fibroblasts, carrying lipids, proteins, and genetic materials, and transmit biological information in vivo [14–17]. According to recent evidence, EVs produced by KLF2-modified ECs (KLF2-EVs) attenuate disease progression, especially in subjects with pulmonary hypertension, atherosclerosis, myocardial ischaemia–reperfusion (I/R) injury and other diseases associated with vascular remodelling [13, 16, 18].

Recently, increasing evidence has indicated that monocyte-derived proinflammatory macrophages dominate the whole pathological process of DOX-induced DCM and lead to cardiac dysfunction [19]. KLF2 EVs inhibit the activation of Mo/Mø and delay the progression of atherosclerosis^16^. As shown in our previous study, KLF2-EVs attenuate myocardial I/R injury by inhibiting Ly6C^high^ monocyte homing to the heart [13]. Based on these results, we established this study to explore the therapeutic potential of KLF2-EVs in the regulation of cardiac inflammation in a DCM model.

## Methods

### Animal experimental protocol

The animal experiments performed in this study were approved by and conducted according to the regulations and guidelines of the Institutional Ethics Committee of Nanjing Drum Tower Hospital (Approval No. 2020AE01081). We purchased 8-week-old C57BL/6 male mice weighing 20–24 g from the Model Animal Research Center of Nanjing University. Animals received a standard laboratory diet and free access to food and water. They were housed in a room with a controlled temperature of 20 °C to 25 °C and humidity of 40% to 70%, with a 12-h light–dark cycle. These mice were randomly divided into the control and DCM groups. Mice in the DCM group received an intraperitoneal injection of doxorubicin (DOX) (20 mg/kg in total) dissolved in saline, whereas mice in the control group were injected with an equal quantity of saline. 7 days after the DOX injection, mice in the DCM group were randomly divided into the DCM + KLF2-EVs group and DCM + PBS group. The DCM + KLF2-EVs group mice were injected with a total of 400 μL PBS containing 200ug KLF2-EVs at 8 μg/g of body weight. The dose of KLF2-EVs was determined according to our preliminary dose-range experiment (Additional file 1: Figure S1).

### Cell culture

Human umbilical vein endothelial cells (HUVECs) were cultured in EC culture medium (Science Cell, 1001) supplemented with 10% foetal bovine serum (FBS, Science Cell, 0025), 1% endothelial cell growth supplement (Science Cell, 1052) and 1% penicillin/streptomycin (Science Cell, 0503). The cultures were maintained at 37 °C with 5% CO2 and 95% humidity. Culture medium was changed every 3 days.

### Recombinant lentivirus vector assembly and transduction into ECs

In this study, we first constructed a GV358 vector (Ubi-MCS-3FLAG-SV40-EGFP-IRES-puromycin) carrying the KLF2 cDNA and then cotransfected it with the lentivirus backbone plasmid into HEK293A cells to produce the recombinant lentivirus vector Lv-KLF2. ECs were cultured at a density of 1 × 10^6^ cells/ml in six-well plates overnight. The lentiviruses (1.5 × 10^9^ TU/ml) were diluted with 1 ml of complete medium containing HitransG P (1 μg/ml, GeneChem, China) and then added to ECs. After 12 h of transfection at 37 °C, the medium was replaced with fresh virus-free medium. After continuous culture for 72 h, we detected successfully transfected cells that presented green fluorescence (GFP-positive) with a fluorescence microscope (I × 53, Olympus Corporation, Japan). Next, puromycin (5 μg/ml) was added to the culture medium to remove negative cells, and the selected cells were KLF2-transfected ECs.

### Exosome isolation and identification

When the ECs grew to 70–80% confluence, the culture medium was replaced with EC culture medium containing 5% EV-depleted FBS, 1% endothelial cell growth supplement and 1% penicillin/streptomycin. Cells were then cultured for 48 h. The EVs were extracted using standard differential centrifugation. Cell culture supernatants were centrifuged at 3,000 g for 25 min and 10,000 g for 1 h at 4 °C to remove dead cells and debris. Subsequently, the supernatants were centrifuged at 100,000 g for 3 h at 4 °C. Finally, the collected EVs were resuspended in phosphate-buffered saline (PBS).

The morphology of exosomes was observed using a transmission electron microscope (JEM-1011 Japan). The number and size were assessed using the NanoSight NS300 system (NanoSight, UK). The EVs were identified by performing western blots for the marker proteins Alix, TSG101, CD63 and CD9. The total protein concentration of EVs was determined using BCA assay (Thermo Scientific).

### Statistical analysis

Quantitative data are presented as the mean ± standard deviation (SD), unless indicated otherwise. Differences in data between groups at a single time point were tested using either two-tailed, unpaired, Student’s t-test or one-way analysis of variance (ANOVA) followed by Tukey’s multiple comparisons test. All analyses were performed using GraphPad Prism 8.0 software (GraphPad Prism Software Inc., San Diego, CA, USA). A p value < 0.05 indicates that the difference was statistically significant.

## Results

### The isolation and identification of KLF2-EVs

**Fig. 1:**
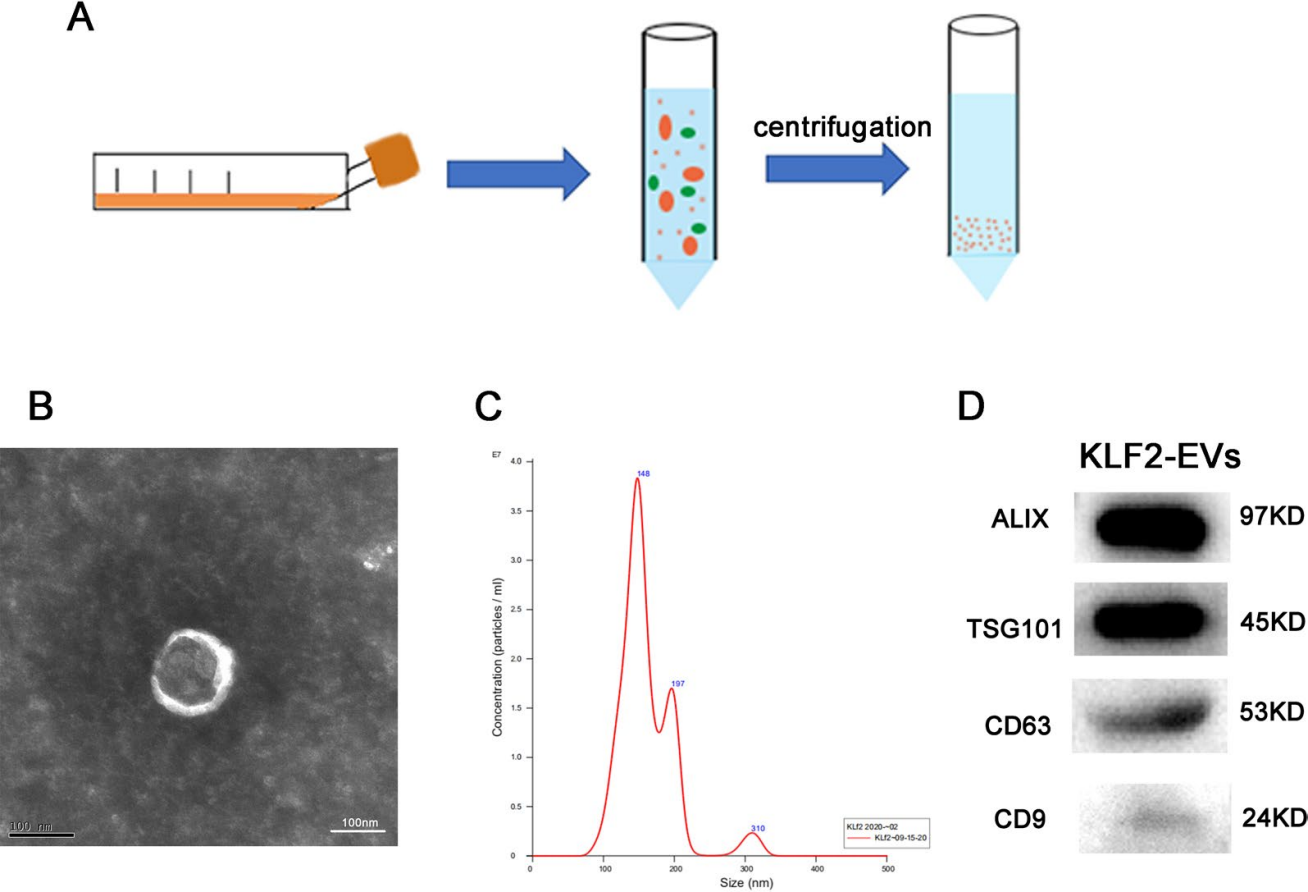
Isolation and characterization of KLF2-EVs. **A** EVs were extracted using gradient centrifugation. **B** The morphology of EVs was analysed using TEM (scale bar, 100 nm). **C** The diameters of isolated EVs were determined using NTA. **D** Representative images of western blots showed the levels of typical EV protein markers, such as ALIX, CD9, CD63 and TSG101

EVs were extracted from the culture supernatants of KLF2-HUVECs through gradient centrifugation (Fig. 1A). We described their morphological and phenotypic characteristics to verify that the isolated vesicles were indeed EVs [20]. First, transmission electron microscopy (TEM) showed that KLF2- EVs had a double-layer membrane structure (Fig. 1B). Then, nanoparticle tracking analysis (NTA) showed that the diameters of the vesicles were within the range of 100–150 nm, with a peak at 148 nm (Fig. 1C). Finally, western blot analysis confirmed that these vesicles expressed ALIX, TSG101, CD63, and CD9, which are recognized as specific membrane proteins for EVs (Fig. 1D).

### KLF2-EVs treatment improved left ventricular function in a mouse DCM model

**Fig. 2:**
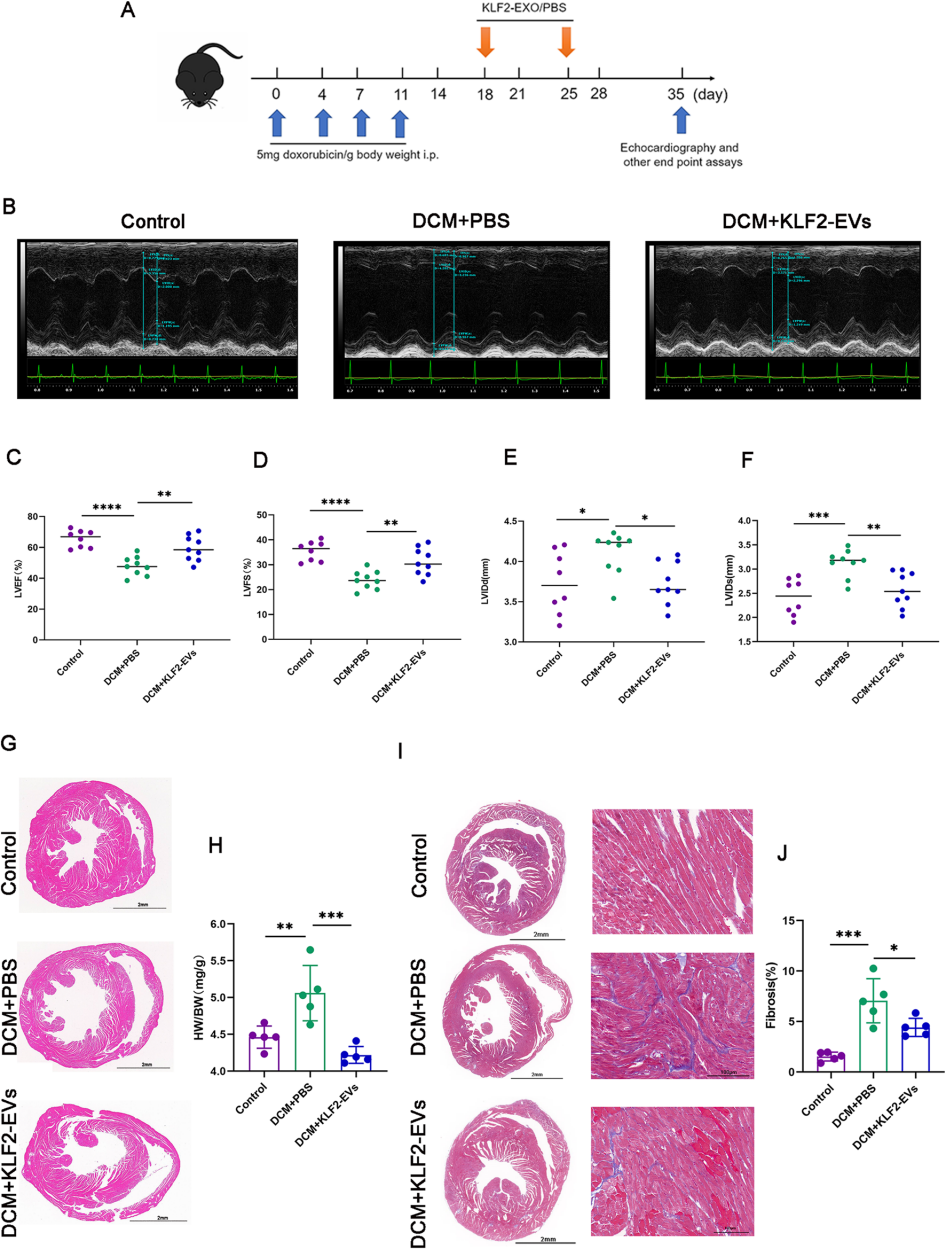
KLF2-EVs improve heart function and ameliorate ventricular remodelling. **A** Mice received repeated low-dose injections of DOX (20 mg/kg in total) and then KLF2-EVs (100 µg of KLF-2 EVs dissolved in 200 µl of PBS) were administered two times via the tail vein. **B** Left ventricular function was assessed using echocardiography (representative short axis view of parasternal M-mode ultrasound). **C** Left ventricular ejection fraction (LVEF, %). **D** Left ventricular fractional shortening (LVFS, %). **E** Left ventricle end-diastolic diameter (LVIDd, mm). **F** Left ventricle end-systolic diameter (LVIDs, mm). **G**–**J** HE (**G**) and MT (**I**) staining were performed on heart sections prepared from mice 5 weeks after the first DOX injection. The ratio of HW/BW (**H**) and quantification of myocardial fibrosis in MT-stained sections (**J**) are shown. *P < 0.05, **P < 0.01, ***P < 0.001, ****P < 0.0001, ns = not significant

Mice received repeated low-dose injections of DOX (20 mg/kg in total) followed by two KLF2-EVs treatments (Fig. 2A). Five weeks after the first DOX injection, echocardiograms were obtained (Fig. 2 and Additional file 1: Table S1 for detailed data). In the DCM + PBS group, LVEF (Fig. 2C) and LVFS (Fig. 2D) were markedly reduced, while LVIDd (Fig. 2E) and LVIDs (Fig. 2F) were increased compared with the control group. Therefore, the animal model of DCM was constructed successfully. Treatment with KLF2-EVs improved LVEF (Fig. 2C) and LVFS (Fig. 2D) and decreased LVIDd (Fig. 2E) and LVIDs (Fig. 2F). Hearts were harvested and stained with Masson’s trichrome (MT) and haematoxylin and eosin (HE) to further explore the effect of KLF2-EVs on cardiac structure. HE staining of heart was shown in Fig. 2G. Compared with the control group, the DCM + PBS group had a higher ratio of HW/BW, while KLF2- EV treatment attenuated these changes (Fig. 2H). Furthermore, MT staining showed that mice in the DCM + KLF2- EV group had less myocardial interstitial fibrosis than mice in the DCM + PBS group (Fig. 2I, J). Therefore, KLF2-EVs exert a protective effect on cardiac function.

### KLF2-EVs reduced myocardial inflammation in mice with DCM

**Fig. 3:**
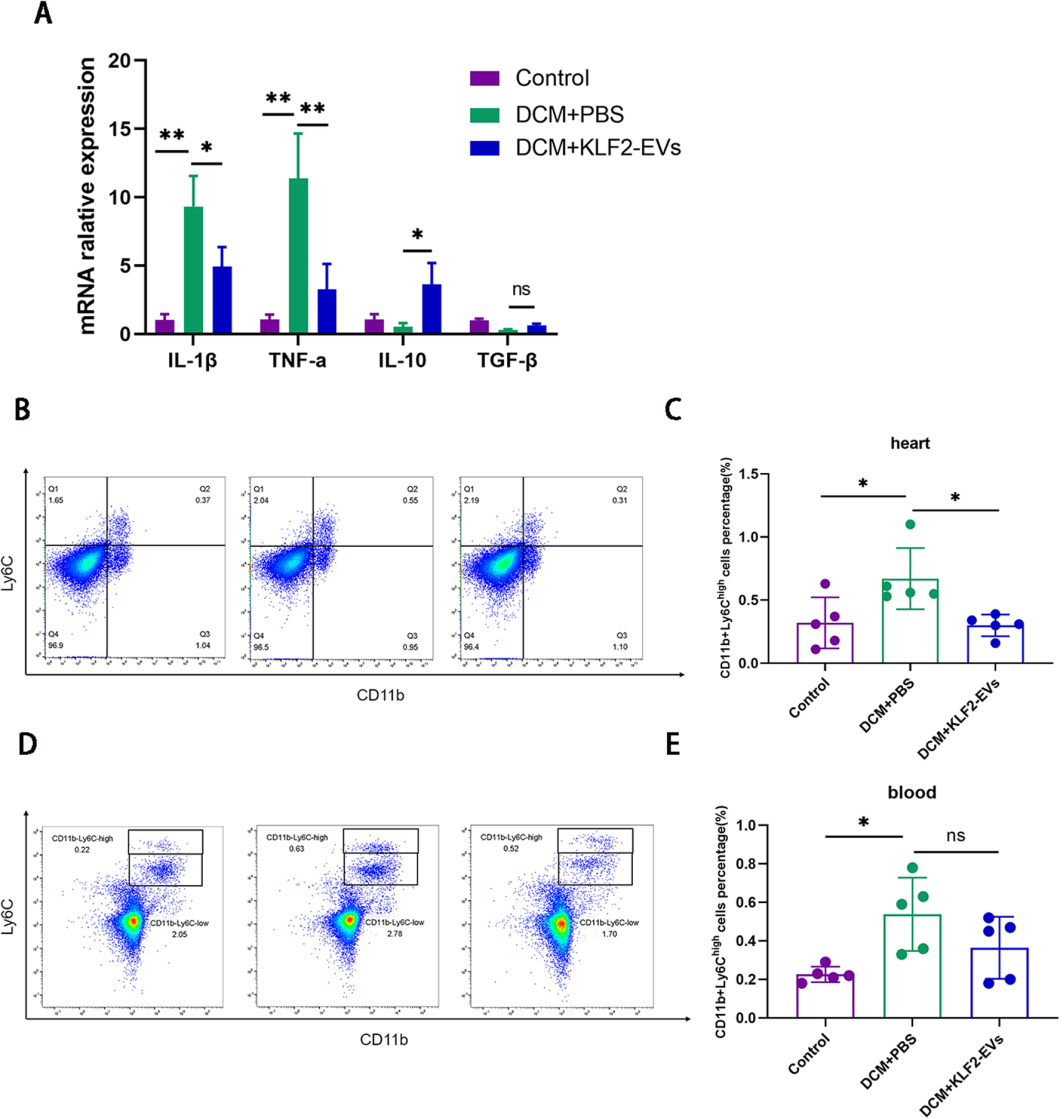
KLF2-EVs reduced the expression of inflammatory factors and decreased Ly6C^high^ Mo/Mø ratios in heart tissue. **A** RT–qPCR analyses of IL-1β、TNF-α 、IL-10 and TGF-β expression. **B** Representative flow cytometry plots showing CD11b + Ly6C^high^ cells in the heart. **C** Quantification of CD11b + Ly6C^high^ cells (Ly6C^high^ Mo/Mø) within the heart tissue. **D** Representative flow cytometry plots showing CD11b + Ly6C^high^ cells in peripheral blood. **E** Quantification of CD11b + Ly6C^high^ cells (Ly6C^high^ Mo/Mø) within blood. *P < 0.05, **P < 0.01, ***P < 0.001, ****P < 0.0001, ns = not significant

The pathogenesis of DCM is associated with chronic intramyocardial inflammation, which is characterized by increased levels of circulating immune cells and inflammatory cytokines^21^. Therefore, we measured the mRNA levels of inflammatory factors in heart tissue using RT–qPCR. Compared with the DCM + PBS group, DCM + KLF2-EVs treatment decreased the mRNA levels of proinflammatory cytokines, including interleukin (IL)-1β and tumour necrosis factor (TNF)-α (Fig. 3A), and increased the levels of anti-inflammatory cytokines, such as IL-10, while transforming growth factor (TGF)-β was slightly increased (Fig. 3A). Since macrophages are a key mediator of DOX-induced myocardial damage, we hypothesized that KLF2-EVs might modulate Mo/Mø responses. Flow cytometry analyses showed that the Ly6C^high^ Mo/Mø ratio in cardiac tissues was increased in the DCM + PBS group. However, KLF2-EVs treatment remarkably reduced the Ly6C^high^ Mo/Mø ratio (Fig. 3B, C). In addition, we analysed monocytes in peripheral blood. KLF2-EVs treatment moderately reduced the number of Ly6C^high^ monocytes, but no significant difference was observed between the DCM + PBS group and the DCM + EVs group (Fig. 3D, E). The results showed that KLF2-EVs treatment reduced the Ly6C^high^ Mo/Mø ratio in the heart tissue.

### KLF2-EVs prevented Ly6C^high^ monocyte recruitment from bone marrow

**Fig. 4:**
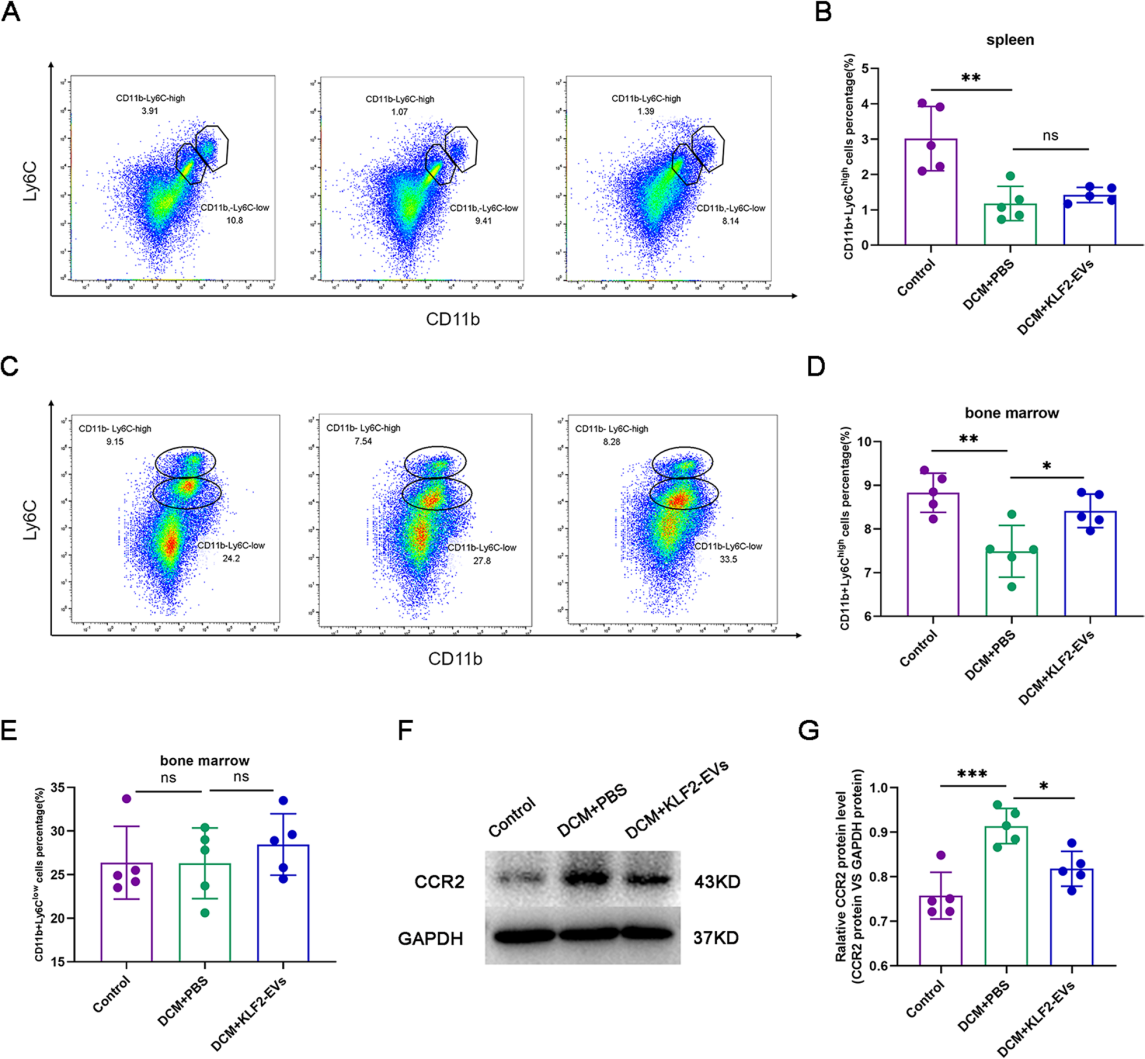
KLF2-EVs prevented Ly6C^high^ monocyte recruitment from bone marrow by inhibiting CCR2 expression. **A** Representative flow cytometry plots showing Ly6C^high^ monocytes (CD11b + Ly6C^high^) in the spleen. **B** Quantification of Ly6C^high^ monocytes in spleen tissues. **C** Representative flow cytometry plots showing Ly6C^high^ monocytes (CD11b + Ly6C^high^) and Ly6C^low^ monocytes (CD11b + Ly6C^low^) in bone marrow. **D**, **E** Quantification of Ly6C^high^ monocytes (**D**) and Ly6C^low^ monocytes in bone marrow. **F** Representative western blot images of CCR2. **G** Quantitative analysis of CCR2 protein levels in bone marrow. Representative images of western blots and quantification of CCR2 levels are shown. *P < 0.05, **P < 0.01, ***P < 0.001, ns = not significant

We observed a decreased Ly6C^high^ Mo/Mø ratio in the hearts of KLF2-EVs-treated mice, but the specific mechanism was unclear. Next, we focused on the inflammatory response after KLF2-EVs treatment. The homing of immune cells, mainly monocytes, from the spleen and bone marrow to the heart occurs after ischaemic heart disease and myocarditis^22^. First, we performed flow cytometry of spleen tissue. No significant change in the Ly6C^high^ Mo/Mø ratio was observed between the DCM + KLF2-EVs group and the DCM + PBS group (Fig. 4A, B). Based on this result, KLF2-EVs did not alleviate cardiac inflammation through a mechanism directly related to the spleen. Next, we assumed that the bone marrow might be involved in the regulation of cardiac inflammation. We extracted cells from the bone marrow and then analysed Ly6C^high^ monocytes (Fig. 4C). We found that the KLF2-EVs treatment increased the retention of Ly6C^high^ monocytes in the bone marrow (Fig. 4D). However, no significant difference in the number of Ly6C^low^ monocytes was observed among the groups (Fig. 4E). Taken together, these results suggested that KLF2-EVs suppressed cardiac inflammatory cells in mice with DCM by restraining the mobilization of Ly6C^high^ monocytes from bone marrow.

Finally, we assessed the possible molecular mechanism of KLF2-EVs and Ly6C^high^ Mo/Mø recruitment in bone marrow. According to previous studies, the migration of mature Ly6C^high^ monocytes is mediated by MCP-1 and CCR2 molecules^23^, ^24^. Here, we extracted cells from bone marrow and observed that a decreased level of the CCR2 protein in the DCM + KLF2-EVs group compared with the DCM + PBS treatment group (Fig. 4F, G).

## Discussion

The main finding of this study is that KLF2-EVs regulate the recruitment of Ly6C^high^ Mo/Mø to the heart and improve cardiac function. This beneficial effect is related to inhibiting the mobilization of Ly6C^high^ monocytes in bone marrow by targeting CCR2 protein expression. Our results suggest that KLF2-EVs may be a potential therapeutic target for the prevention and treatment of DCM.

ECs are critical due to their vascular anti-inflammatory and antithrombotic activities [25, 26]. KLF2 is activated by shear stress and plays a key role in the development of the lung [18, 27]. Studies have indicated that KLF2 blocks the transduction of proinflammatory signalling in ECs [11]. EVs are biological vesicles with membrane structures that are secreted by cells, and they are essential mediators of intercellular information transmission and regulation of cell function [28–32]. EVs secreted by ECs regulate the activation of monocytes or the phenotype of macrophages^16^, ^30^. Additionally, changes in the amount of EVs released might affect vascular inflammation in cardiovascular diseases [31, 33]. Recently, the development of pulmonary hypertension was reported to be associated with reduced KLF2 signalling, and KLF2-regulated EVs play important regulatory roles in vascular remodelling and vascular homeostasis [18]. Although the beneficial effect of KLF2-EVs on cardiovascular disease has been identified, the underlying functions and mechanisms in DCM have not been extensively studied.

In the present study, a mouse DCM model was established by intraperitoneally injecting DOX to simulate the formation of human DCM. The KLF2-EVs treatment improved cardiac function and inhibited ventricular remodelling. In addition, inflammatory cells, especially macrophages, exert an essential effect on the pathogenesis of DCM. Previous studies have shown an increased number of macrophages in human and mouse heart tissues with DCM [34, 35]. In DOX-induced cardiomyopathy, resident macrophages exhibit increased proliferation and confer a reparative role, while monocyte-derived macrophages primarily exhibit a proinflammatory phenotype that dominates the whole DCM [19]. Therefore, we overexpressed KLF2 to mimic the physiological and anti-inflammatory phenotype of cultured human umbilical vein endothelial cells (HUVECs). Here, we found that KLF2-EVs increased the levels of anti-inflammatory factors and reduced the levels of proinflammatory factors. Previous studies also showed that exosomes derived from mouse coronary endothelial cells exert anti-inflammatory effects [13]. Furthermore, KLF2-EVs reduced the Ly6C^high^ Mo/Mø ratio in myocardial tissue and peripheral blood, but the difference in peripheral blood was not significant. A previous study reported significantly improved heart function in a mouse model of doxorubicin-induced DCM after removing monocyte-derived macrophages by injecting clodronate liposomes [19]. Therefore, KLF2-EVs exert cardioprotective effects by regulating the Mo/Mø system in DCM.

Cardiac-resident Mo/Mø are relatively limited in number. Therefore, most Mo/Mø are recruited from the periphery when inflammation occurs in myocardial tissue. Next, we speculated that the possible mechanism by which myocardial Ly6C^high^ Mo/Mø ratios are reduced is that KLF2-EVs inhibited the mobilization and recruitment of Ly6C^high^ Mo/Mø. Cardiac proinflammatory macrophages are mainly derived from the differentiation of peripheral monocytes. According to recent studies, the locally accumulated Ly6C^high^ Mo/Mø in the heart were mainly derived from the spleen and bone marrow after myocardial I/R injury [36]. At the beginning of inflammation, Mo/Mø are mobilized from the spleen tissue [37]. Then, mature Ly6C^high^ monocytes mobilizing from bone marrow enter the blood and are recruited to the heart. In the present study, KLF2-EVs treatment did not affect the Ly6C^high^ Mo/Mø ratio in the spleen but increased the retention of Ly6C^high^ Mo/Mø in bone marrow. Mature Ly6C^high^ monocyte migration from bone marrow to blood and from blood to heat is mediated by MCP-1/CCR2[38]. The expression of the CCR2 protein decreased after KLF2-EVs treatment, suggesting that KLF2-EVs effectively inhibited CCR2 expression. Taken together, our findings suggested that KLF2-EVs inhibited the mobilization of Ly6C^high^ Mo/Mø in the bone marrow by inhibiting CCR2 protein expression. This finding provides a potential approach for treating DCM.

Our study has some limitations. The pathogenesis of DCM is complicated and related to many factors [39, 40]. Thus, establishing an ideal animal model is crucial for studying the pathogenesis of DCM. We constructed a mouse DCM model by injecting DOX intraperitoneally. DOX damages cardiomyocytes, and local fibrosis gradually replaces the injured myocardial tissue, which causes similar pathophysiological changes to DCM and subsequent congestive heart failure [41]. However, this method does not fully simulate various forms of DCM. In addition, the main sites in which EVs accumulate are the liver, lungs, gastrointestinal tract, and spleen. We must inject a large number of EVs to confirm that sufficient numbers of EVs reached the target to exert their effects. Finally, multiple types of microRNAs are carried in EVs, and microRNAs that play a major role in the development of DCM remain to be further studied.

## Conclusions

We show here that KLF2-transduced EC-derived EVs improve cardiac systolic function and reduce ventricular remodelling in mice with DOX-induced DCM. This effect may be mediated by downregulating the expression of the CCR2 protein, thereby inhibiting the mobilization of ly6C^high^ monocytes in bone marrow. This study provides a new therapeutic approach for DCM.

## Supplementary Information

**Additional file 1: Table 1**. Echocardiographic parameters after KLF2-EVs treatment.

## Data Availability

All data used to generate these results are available in the main text and supporting information.
