# Supplementary material for: The effects of extracellular vesicles derived from Krüppel-Like Factor 2 overexpressing endothelial cells on the regulation of cardiac inflammation in the dilated cardiomyopathy
Source: J Nanobiotechnology. 2022 Feb 9;20:76. doi: 10.1186/s12951-022-01284-1 (PMC8827179; doi:10.1186/s12951-022-01284-1)
Supplement: Supplementary file 1 — Additional file 1: Table 1. Echocardiographic parameters after KLF2-EVs treatment. [file 12951_2022_1284_MOESM1_ESM.docx]

**The effects of extracellular vesicles derived from Krüppel-Like Factor 2 overexpressing endothelial cells on the regulation of cardiac inflammation in the dilated cardiomyopathy**

Wenfeng Zhang ^1#^, Ziwei Chen ^1#^, Shuaihua Qiao^2#^, Siyuan Chen^1^; Hongyan Zheng ^2^, Xuan Wei ^2^, Qiaoling Li^3*^, Biao Xu^4,5*^, Wei Huang ^6*^

^1^ Department of Cardiology, Nanjing Drum Tower Hospital Clinical College of Nanjing Medical University, Nanjing, 210008, China.

^2^ Department of Cardiology, Affiliated Drum Tower Hospital, Medical School of Nanjing University, Nanjing, 210008, China.

^3^ Department of Cardiology, Affiliated Drum Tower Hospital, Medical School of Nanjing University, Nanjing, 210008, China. [lqldoctor@126.com](mailto:lqldoctor@126.com).

^4^ Department of Cardiology, Nanjing Drum Tower Hospital Clinical College of Nanjing Medical University, Nanjing, 210008, China. [xubiao62@nju.edu.cn](mailto:xubiao62@nju.edu.cn).

^5^ Department of Cardiology, Affiliated Drum Tower Hospital, Medical School of Nanjing University, Nanjing, 210008, China. [xubiao62@nju.edu.cn](mailto:xubiao62@nju.edu.cn).

^6^ Department of Cardiology, Nanjing Drum Tower Hospital Clinical College of Nanjing Medical University, Nanjing, 210008, China. 1210252287@qq.com.

^#^ These authors contributed equally to this work.

^*^ These authors contributed equally to this work.

**SUPPLEMENTAL METHODS**

Echocardiography

5 weeks after the first DOX injection, mice were under light anesthesia and two-dimensional short and long axes imaging were acquired to calculate LV functional parameters. LV end-systolic diameter (LVID; d), LV end-diastolic diameter (LVID; s), interventricular septal thickness (IVS) and LV posterior wall thickness (LVPW) (end-diastolic and end-systolic) were measured from at least three consecutive cardiac cycles on the M-mode tracings. LV fractional shortening (FS %) was determined as [(LVID;d– LVID; s)/LVID; d]× 100. LV ejection fraction (EF) was calculated as: EF (%) = ((LV Vol; d-LV Vol; s)/LV Vol; d) × 100. LV Vol; d = ((7.0 / (2.4 + LVID; d)) × LVID;d3); LV Vol; s = ((7.0 / (2.4 + LVID;s)) × LVID;s3).

Histological analysis

Histology of hearts was assessed 5 weeks after first doxorubicin injection. We collected the hearts in diastole late term with intraventricular injection of 10% potassium chloride (KCl).

The heart was fixed with 4% phosphate-buﬀered formalin (pH 7.4). Then the tissues were dehydrated by gradient ethanol and set in paraffin which cut into 5‐μm sections. Hematoxylin-eosin (H.E.) was applied to analyze the global heart morphology. and Masson trichrome (MT) to identify cardiac fibrosis. Quantification of cardiac left ventricular area and fibrosis was calculated by ImageJ.

Flow cytometry analysis

Isolated cell suspensions from heart, spleen, bone marrow and peripheral blood were obtained by gentle MACS™ Dissociator (Miltenyi Biotec). Then the samples were incubated with CD11b‐FITC (BD Bioscience) and Ly6C-PE (BD Bioscience) for 30 minutes at 4°C. Data from FACS Aria flow cytometer (BD Bioscience) were analyzed with FlowJo software. We used CD11b and Ly6C to describe monocyte phenotype subset and used percentage to evaluate Ly6C^high^ and Ly6C^low^ monocyte/macrophages (Mo/Mø). For example, within heart samples, percentage was the Ly6C^high^ Mo/Mø numbers to all living cardiac cells numbers, and within peripheral blood samples, percentage was the Ly6C^high^ monocytes numbers to all blood living cells numbers.

RNA extraction and reverse‐transcription quantitative polymerase chain reaction(RT-qPCR)

Total RNA was extracted from heart tissue following Trizol (Invitrogen) according to the manufacturer’s instructions. Then cDNA was synthesized by the HiScript III RT SuperMix for qPCR (+gDNA wiper) (Vazyme) and qPCR was carried out by ChamQ SYBR qPCR Master Mix (High ROX Premixed) (Vazyme). We used 2−ΔCt to calculate relative gene expression level and data were normalized to GAPDH expression. All the primers involved were as follows:

| Primer | sequence |
| --- | --- |
| GAPDHforward | TGTGTCCGTCGTGGATCTGA |
| GAPDH reverse | TTGCTGTTGAAGTCGCAGGAG |
| TNFα forward | CCCCAAAGGGATGAGAAGTTC |
| TNFα reverse | GCTTGTCACTCGAATTTTGAGAA |
| IL-1β forward | TGAAGTTGACGGACCCCAAA |
| IL-1β reverse | TGATGTGCTGCTGTGAGATT |
| TGFβ1 forward | GAAGGACCTGGGTTGGAAGTGGATC |
| TGFβ1 reverse | TGTGTTGGTTGTAGAGGGCAAGGAC |
| IL-10 forward | GTTGCCAAGCCTTATCGGGAA |
| IL-10 reverse | CCAGGGAATTCAAATGCTCCT |
| Collagen 2 forward | ACCCTTCTCACTCCTGAAGGCTCTA |
| Collagen 2 reverse | TATGAGTTCTTCGCTGGGGTGTTTA |
| Collagen 3 forward | TGCCATTGCTGGAGTTGGA |
| Collagen 3 reverse | GAAGACATGATCTCCTCAGTGTTGA |

Western blot

The total protein was extracted from bone marrow tissue and KLF2-EVs. Samples were lysed in cold lysis buffer with protease inhibitor (KEYGEN BIOTECH). 30µg of each protein sample was separated by SDS-PAGE gel (10%) and then transferred to PVDF membrane (Millipore, Bedford, MA, USA). After blocking with 5% bovine serum albumin, the blots were probed with a primary antibody (1:1000) followed by a horseradish peroxidase-conjugated secondary antibody (1:5000). The following primary antibodies were used: CCR2 (Abcam, ab203128), GAPDH (MultiSciences, ab011), CD9 (Abcam, ab92726), CD63 (Abclonal, A5271), TSG101 (Proteintech, 28283-1-AP), Alix (Abcam, ab117600). The secondary antibody used were HRP-labeled Goat Anti-rabbit IgG(H+L) (Beyotime, A0208) and HRP-labeled Goat Anti-mouse IgG(H+L) (Beyotime, A0216). Enhanced chemiluminescence (ECL, thermofisher scientific) was used to detect the protein levels on the blots.

SUPPLEMENTAL FIGURES

**
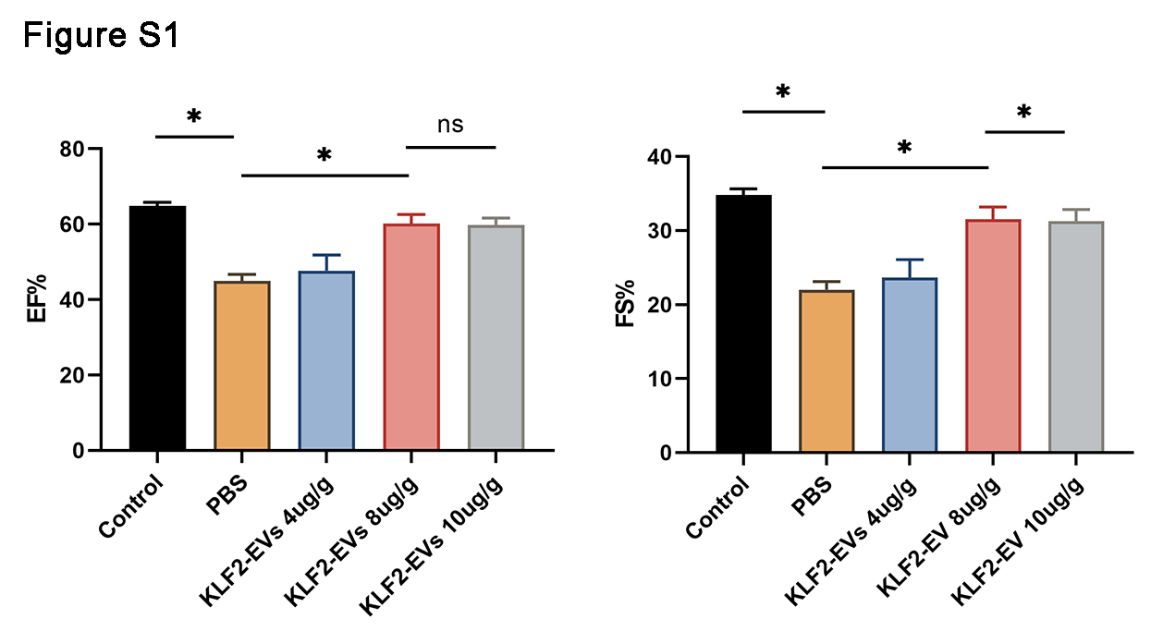
**

**
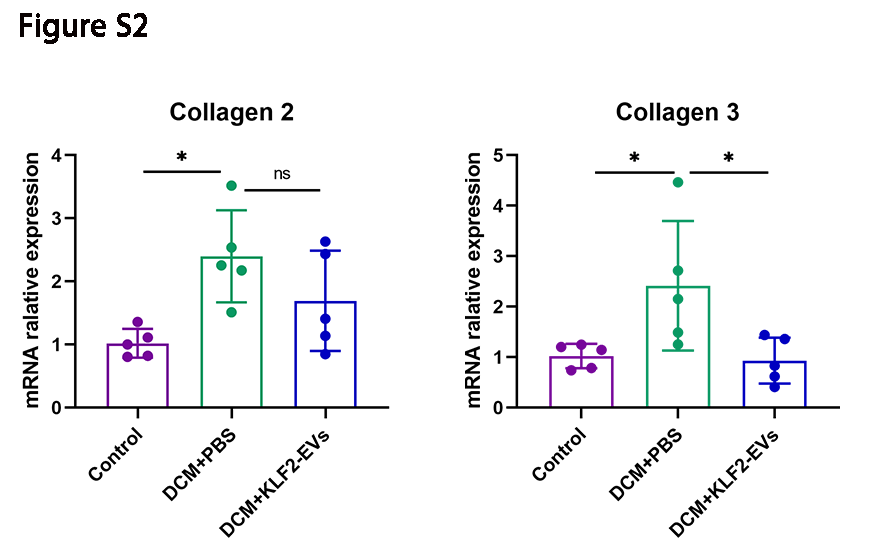
**

SUPPLEMENTAL TABLES

Supplemental Table 1 Echocardiographic parameters after KLF2-EVs treatment

|  | **Control**  **(n=8)** | **DCM+PBS**  **(n=9)** | **DCM+KLF2- EXO (n=9)** |
| --- | --- | --- | --- |
| **LVEF (%)** | 65.57±5.55 | 47.91±6.13^****^ | 59.52±8.17^##^ |
| **LVFS (%)** | 35.49±1.38 | 23.90±3.65^****^ | 31.27±5.50^##^ |
| **LVIDd (mm)** | 3.73±0.39 | 4.11±0.26^*^ | 3.73±0.26^#^ |
| **LVIDs(mm)** | 2.42±0.38 | 3.12±0.28^***^ | 2.58±0.36^##^ |
| **LVPWd(mm)** | 0.72±0.07 | 0.67±0.10 | 0.72±0.03 |
| **LVPWs(mm)** | 1.15±0.11 | 0.94±0.16^**^ | 1.11±0.10^#^ |
| **IVSTd(mm)** | 0.73±0.06 | 0.71±0.07 | 0.73±0.04 |
| **IVSTs(mm)** | 1.21±0.12 | 1.03±0.06^**^ | 1.13±0.09 |
| **LV Vol;s (uL)** | 21.33±9.08 | 40.03±8.62^*^ | 24.80±8.32^#^ |
| **LV Vol;d (uL)** | 60.24±11.34 | 76.52±12.66^***^ | 59.83±9.99^##^ |
| **LV Mass (mg)** | 91.71±14.78 | 103.68±14.95 | 92.69±12.35 |
| **LV Mass**  **(Corrected)(mg)** | 73.37±7.97 | 82.94±11.96 | 74.15±9.88 |

LVEF, left ventricular ejection fraction; LVFS, left ventricle fractional shortening; LVIDs, left ventricle end-diastolic diameter ;LVIDs, left ventricle end-systolic diameter; LVPWd, left ventricular posterior wall thickness at end-diastole; LVPWs, left ventricular posterior wall thickness at end- systole; IVSTd, interventricular septal thickness at end-diastole; IVSTs, interventricular septal thickness at end- systole; LV Vol;d, left ventricle end diastolic volume; LV Vol;s, left ventricle end systolic volume; LV Mass, left ventricular heart weight ; LV Mass (Corrected): left ventricular heart weight(Corrected). **P* < 0.05，***P* < 0.01，****P* < 0.001，*****P* < 0.0001 vs. Control。#*P* < 0.05，##*P* < 0.01 vs. DCM+PBS.
